# Rediscovery of the Threatened River Sharks, *Glyphis garricki* and *G*. *glyphis*, in Papua New Guinea

**DOI:** 10.1371/journal.pone.0140075

**Published:** 2015-10-07

**Authors:** William T. White, Sharon A. Appleyard, Benthly Sabub, Peter M. Kyne, Mark Harris, Rickson Lis, Leontine Baje, Thomas Usu, Jonathan J. Smart, Shannon Corrigan, Lei Yang, Gavin J. P. Naylor

**Affiliations:** 1 Australian National Fish Collection, National Research Collections Australia, Commonwealth Scientific and Industrial Research Organisation, Hobart, Tasmania, Australia; 2 Oceans & Atmosphere, Commonwealth Scientific and Industrial Research Organisation, Hobart, Tasmania, Australia; 3 National Fisheries Authority, National Capital District, Port Moresby, Papua New Guinea; 4 Research Institute for the Environment and Livelihoods, Charles Darwin University, Darwin, Northern Territory, Australia; 5 F.F.C. Elasmobranch Studies, New Port Richey, Florida, United States of America; 6 Centre for Sustainable Tropical Fisheries and Aquaculture & School of Earth and Environmental Sciences, James Cook University, Townsville, Queensland, Australia; 7 Department of Biology, College of Charleston, Charleston, South Carolina, United States of America; University of Minnesota, UNITED STATES

## Abstract

Recent surveys of the shark and ray catches of artisanal fishers in the Western Province of Papua New Guinea (PNG) resulted in the rediscovery of the threatened river sharks, *Glyphis garricki* and *Glyphis glyphis*. These represent the first records of both species in PNG since the 1960s and 1970s and highlight the lack of studies of shark biodiversity in PNG. Two individuals of *G*. *garricki* and three individuals of *G*. *glyphis* were recorded from coastal marine waters of the Daru region of PNG in October and November 2014. The two *G*. *garricki* specimens were small individuals estimated to be 100–105 cm and ~113 cm total length (TL). The three *G*. *glyphis* specimens were all mature, one a pregnant female and two adult males. These are the first adults of *G*. *glyphis* recorded to date providing a more accurate maximum size for this species, i.e. ~260 cm TL. A single pup which was released from the pregnant female *G*. *glyphis*, was estimated to be ~65 cm TL. Anecdotal information from the fishers of pregnant females of *G*. *glyphis* containing 6 or 7 pups provides the first estimate of litter size for this species. The jaws of the pregnant female *G*. *glyphis* were retained and a detailed description of the dentition is provided, since adult dentition has not been previously documented for this species. Genetic analyses confirmed the two species cluster well within samples from these species collected in northern Australia.

## Introduction

Papua New Guinea (PNG) sits within the Coral Triangle, a region of exceptional marine biodiversity. Despite being key components of this biodiversity and marine ecosystems more broadly, there is a paucity of information on sharks in PNG waters with much of our knowledge coming from historical, rather than contemporary, records scattered across a wide range of scientific publications and expedition reports (e.g. [1–3]). The lack of even the most fundamental biodiversity information hinders a proper assessment of the impacts of the various pressures exerted on sharks in the region, e.g. fishing, pollution from mining, habitat loss. In recognition of this lack of detailed data on the shark and ray resources of PNG, the National Fisheries Authority in Port Moresby has initiated a large-scale project to obtain detailed data on the biodiversity and utilisation of elasmobranchs (sharks and rays) in its national waters.

The river sharks (Carcharhinidae: *Glyphis*) are a relatively poorly known group of sharks with patchy distributions in tropical rivers and coastal regions of the Indo-West Pacific. Two species with sympatric distributions are known from the Australian-New Guinea region; the relatively recently described Northern River Shark *Glyphis garricki* Compagno, White & Last and the Speartooth Shark *Glyphis glyphis* (Müller & Henle) [4]. In PNG waters, both species are known from very few records. *Glyphis garricki* records are based on jaws collected from the Gulf of Papua off Port Romilly (07°40′ S, 144°50′ E) in 1966 and Baimuru (07°33′ S, 144°51′ E) in 1974. *Glyphis glyphis* records are also based on jaws collected from the Gulf of Papua off Port Romilly (07°40′ S, 144°50′ E) in 1966 and Alligator Island (07°19′ S, 141°11′ E), date unknown.

In northern Australia, *G*. *glyphis* has been recorded from nine tidal rivers and estuaries, all of which are highly turbid with fine muddy substrates in salinities of 0.8–28.0 [5]. In the Adelaide River of the Northern Territory, individuals have been found up to 100 km inland with larger individuals occurring closer to the river mouth and smaller juveniles 80–100 km upstream during the late dry season [5]. No adult specimens of this species have been previously reported. *Glyphis garricki* has been recorded in several large tidal tropical river systems and coastal habitats also characterised by fine muddy substrates and high turbidity [5]. Juveniles and subadults are found in freshwater, estuarine and marine environments (salinities 2–36), whilst adults have only been recorded from marine areas [5]. In northern Australia, the presence of free-swimming neonates in October suggests that both *G*. *glyphis* and *G*. *garricki* give birth in October [5].

During surveys of shark and ray catches of artisanal fishing activities in the Daru region of PNG, two individuals of *G*. *garricki* and three individuals of *G*. *glyphis* were recorded. These represent the first confirmed records of *Glyphis* species in PNG waters since the 1960s and 1970s. The *G*. *glyphis* specimens were all adults, one a pregnant female from which only the jaws and fins were observed and two adult males based on images taken by fishers from Katatai. This represents the first adult specimens of *G*. *glyphis* recorded. The dentition of the adult female *G*. *glyphis* specimen is described in detail and insights into the ecology of the species are discussed, including a first account of litter size of *G*. *glyphis*. The two *G*. *garricki* were juvenile specimens. Species identification of the *Glyphis* specimens was confirmed using molecular analyses.

## Materials and Methods

### Ethics Statement

The adult female *Glyphis glyphis* examined in this study, consisting of its jaws and most fins, was captured by artisanal gillnet fishers from the village of Katatai (9°01’15” S, 143°20’31” E) in October 2014. The fins and jaws were brought to Daru the following morning for sale. The gill nets used are ~2 m high and ~100 m long and have up to 9 inch mesh size, and at this time of the year were set relatively close to shore in shallow water. The target species for this fishery at this time of the year (October) is the Barramundi *Lates calcarifer* (Bloch). The two adult male *G*. *glyphis* and one specimen of *G*. *garricki* were caught by the same fishers in the following month (November 2014) and images were supplied to the project team from the village chairman (J. Page). A second specimen of *G*. *garricki*, consisting of only the dried first dorsal fin, was observed at the fish buyer company Philo Marine Ltd. in Daru where dried shark fin and fish swim bladders are exported. For all specimens recorded, death occurred following entanglement in the gillnet and each were dead when the nets were retrieved by the fishers.

Permission was obtained from Philo Marine Ltd. to examine their dried shark fins, which included the *G*. *garricki* fin. Permission was obtained to examine the female *G*. *glyphis* fins and jaw, the latter of which was subsequently purchased from the Katatai village chairman to be retained as a museum specimen. Although *G*. *garricki* and *G*. *glyphis* are listed as Endangered and Critically Endangered, respectively, on the Australian *Environment Protection and Biodiversity Conservation Act*, and Critically Endangered [6] and Endangered [7], respectively, on the IUCN Red List of Threatened Species, there is currently no regulation against taking of these species in PNG waters. Approval from CSIRO or National Fisheries Authority for research using moribund bycatch from fishers is not required.

The artisanal gillnet fishers are permitted to fish in the Daru region by the National Fisheries Authority. The commercial fish buyer (Philo Marine Ltd.) is certified to export dried fish products from Daru under the Independent State of Papua New Guinea’s *Companies Act 1997*.

### Specimens Examined

**Fig 1:**
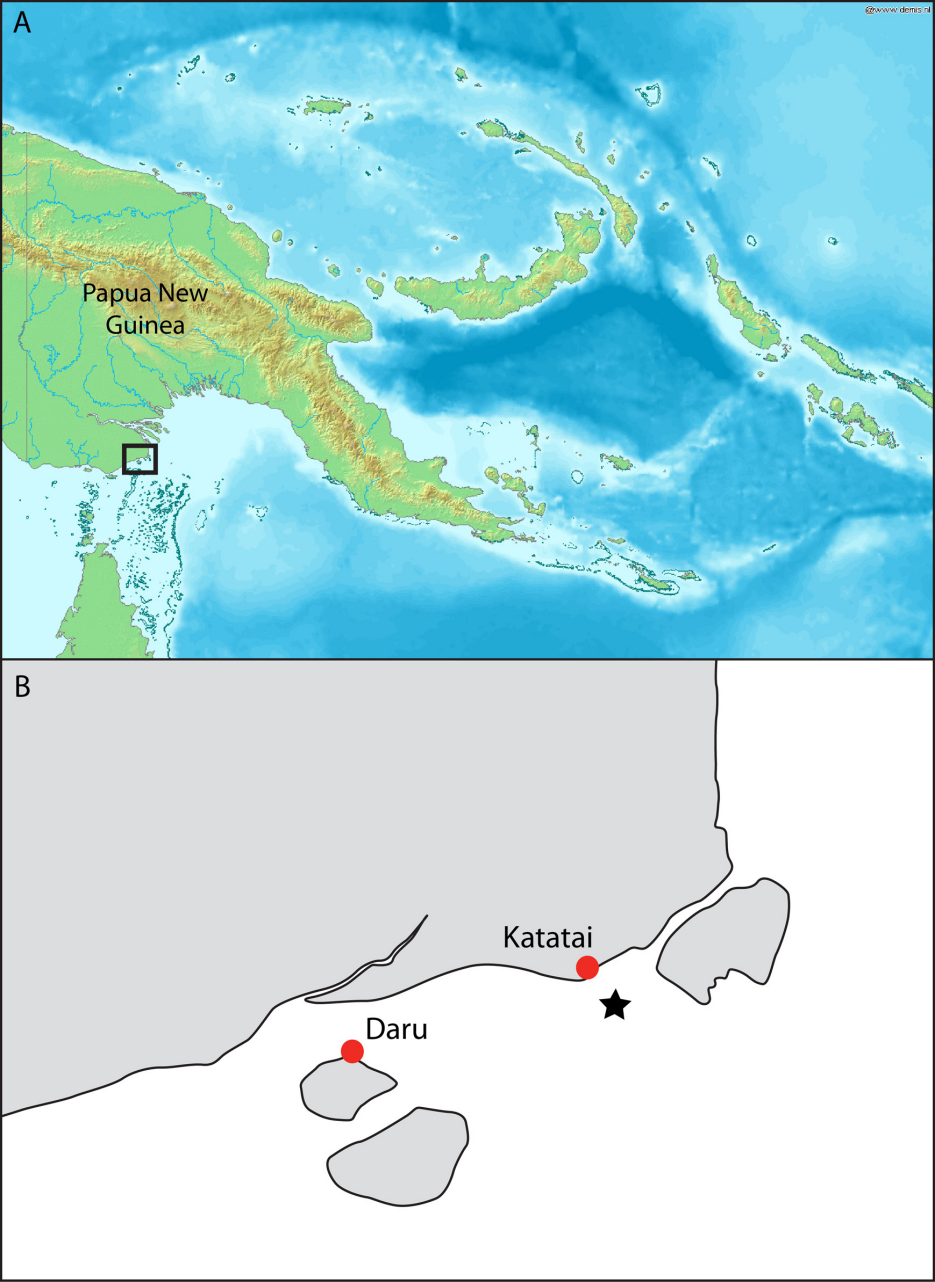
Map of Papua New Guinea and the Daru region. (A) Papua New Guinea with the black box indicating the Daru region; (B) Inset of the Daru region from where the five *Glyphis* specimens were caught by fishers from the village of Katatai. Black star indicates the approximate capture location of the pregnant female *Glyphis glyphis*.

**Fig 2:**
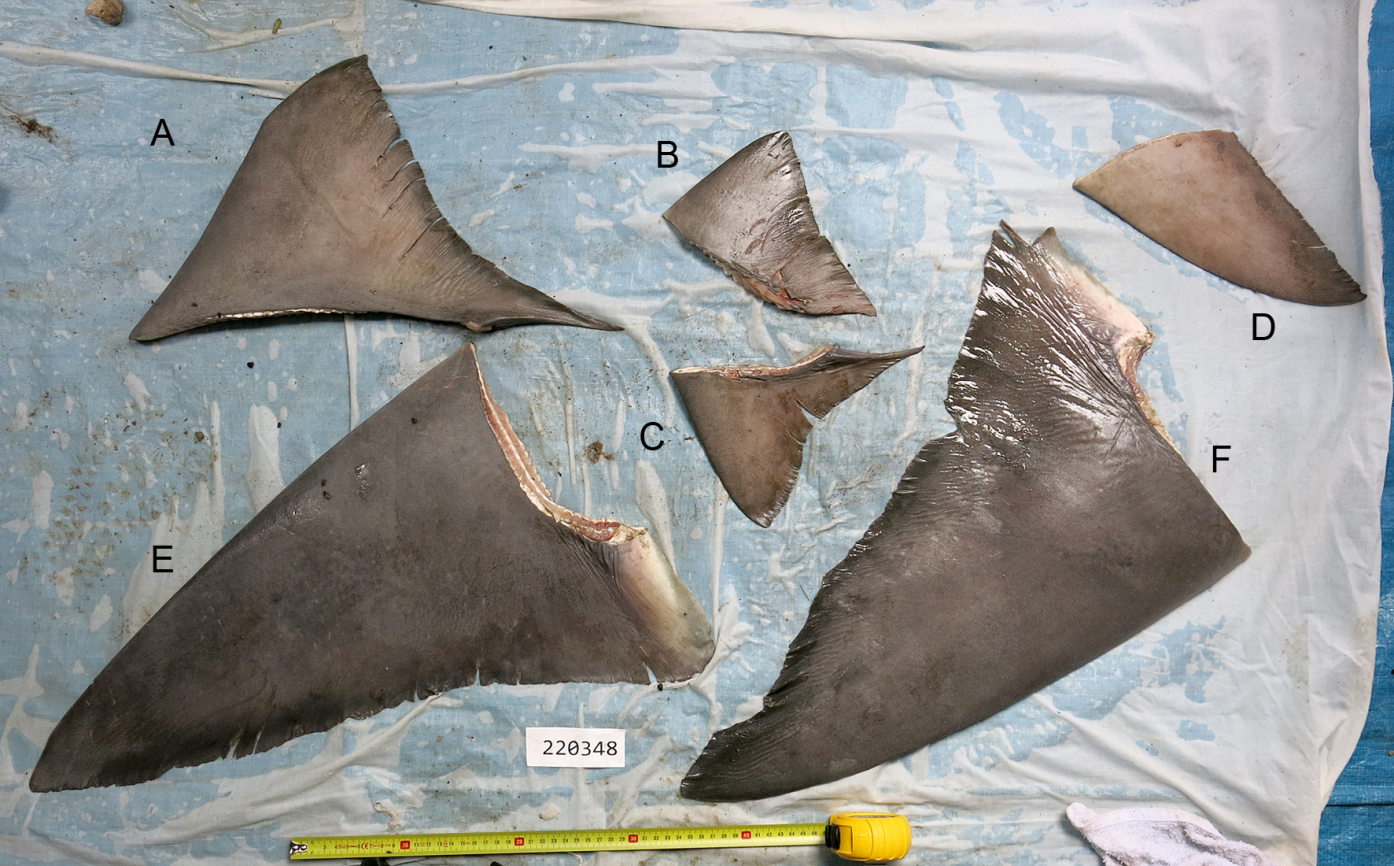
Fresh fins of the adult female *Glyphis glyphis*. Field code 220348, estimated length 237–260 cm: (A) first dorsal fin; (B) right pelvic fin; (C) second dorsal fin; (D) lower caudal-fin lobe; (E) left pectoral fin; (F) right pectoral fin. Tape measure is set at 47 cm.

**Fig 3:**
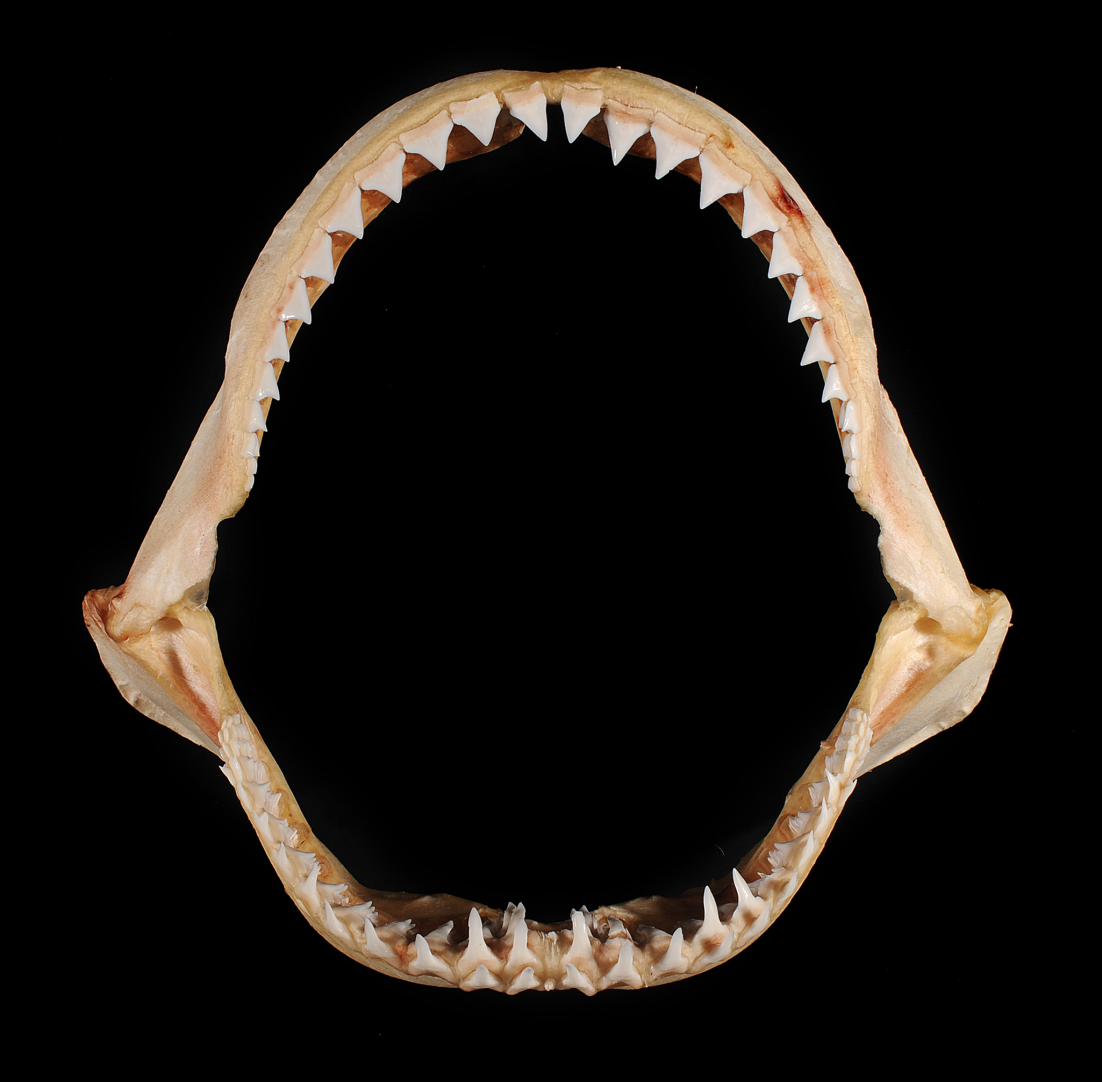
Whole jaw of *Glyphis glyphis*. Adult female (CSIRO H 7670–01).

During a field trip to the island of Daru (9°03’55” S, 143°12’35” E) in the Western Province of PNG (Fig 1) in October 2014, a number of fishing villages and camps were visited along the mainland coast. At each of these villages or camps, the local chairman was informed of our study and asked to bring any captured sharks and rays to the market area in Daru over the week of the survey trip. On the 23^rd^ October 2014, the chairman from the village of Katatai (9°01’15” S, 143°20’31” E, Fig 1) brought several shark and ray specimens to Daru. Included in these specimens were the lower caudal lobe, dorsal fins, pectoral fins, one pelvic fin, and jaw of a large *G*. *glyphis* specimen that was caught in a gillnet set about 3 km offshore from the fishing village of Katatai in marine waters (Fig 1). The fins were photographed and the pectoral and first dorsal fin measured (Fig 2). The jaw was retained and is deposited in the Australian National Fish Collection in Hobart (accession number CSIRO H 7670–01; Fig 3). A muscle tissue sample was taken from the jaws and stored in 100% ethanol. The majority of the flesh and connective tissue was removed from the jaws and which was then dried completely in a fume cupboard.

**Fig 4:**
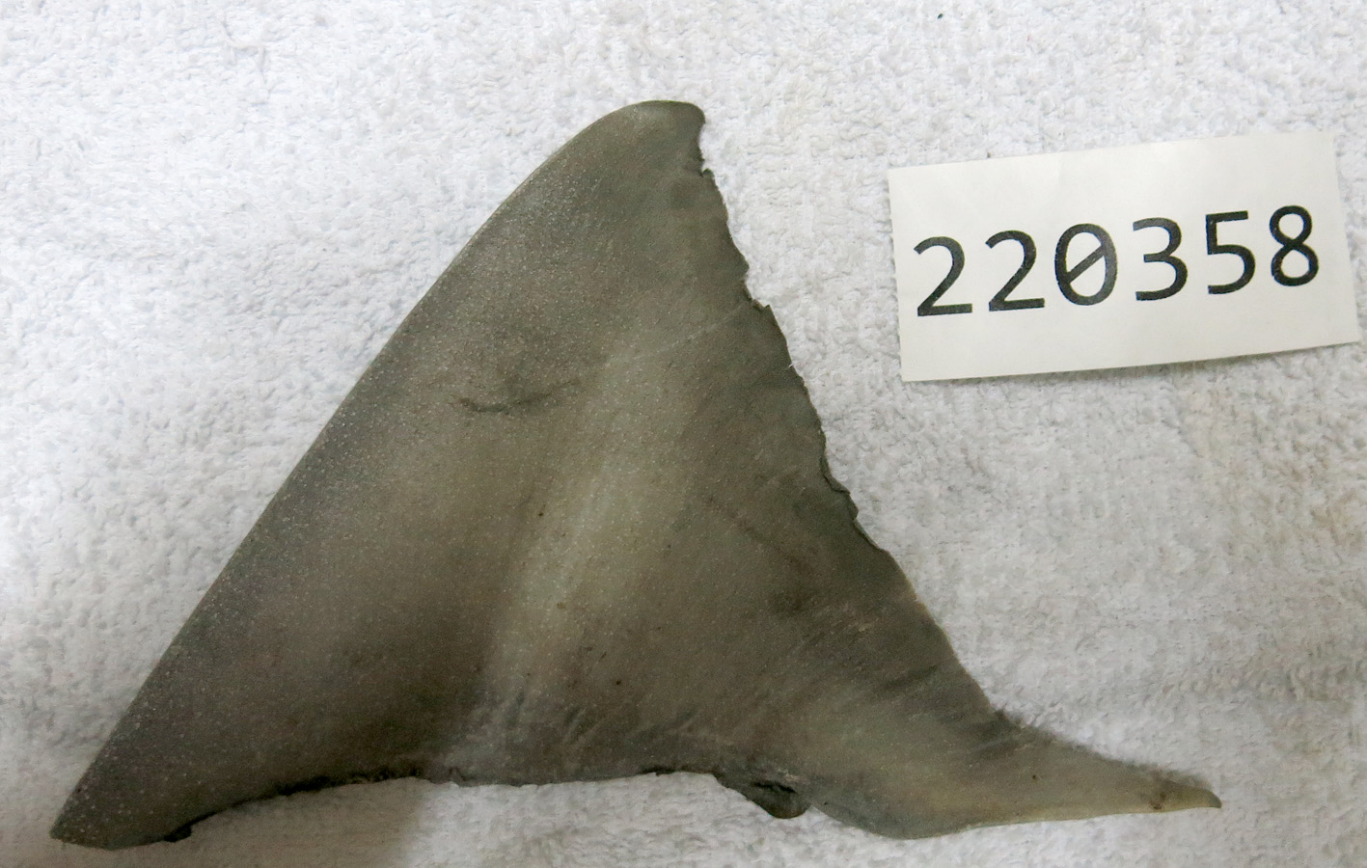
Dried first dorsal fin of *Glyphis garricki*. Field code 220358, estimated length 100–105 cm.

A batch of dried shark fins present at the fish buyer company Philo Marine Ltd. in Daru was examined on the 25^th^ October 2014. In order to determine the number of shark specimens present in the batch of dried fins, all first dorsal fins were separated from the remaining fins. For sawfish, guitarfish and wedgefish which have two similar-sized dorsal fins, the caudal fin was used to prevent duplication of specimens. It was determined that 66 specimens of sharks and rays were present in the batch of dried fins. For each of the selected first dorsal fins, an image, fin measurements and a small piece of tissue from the free rear tip were taken. Subsequent DNA barcoding techniques revealed that one of the 66 fins was *G*. *garricki* (see molecular section below; Fig 4).

**Fig 5:**
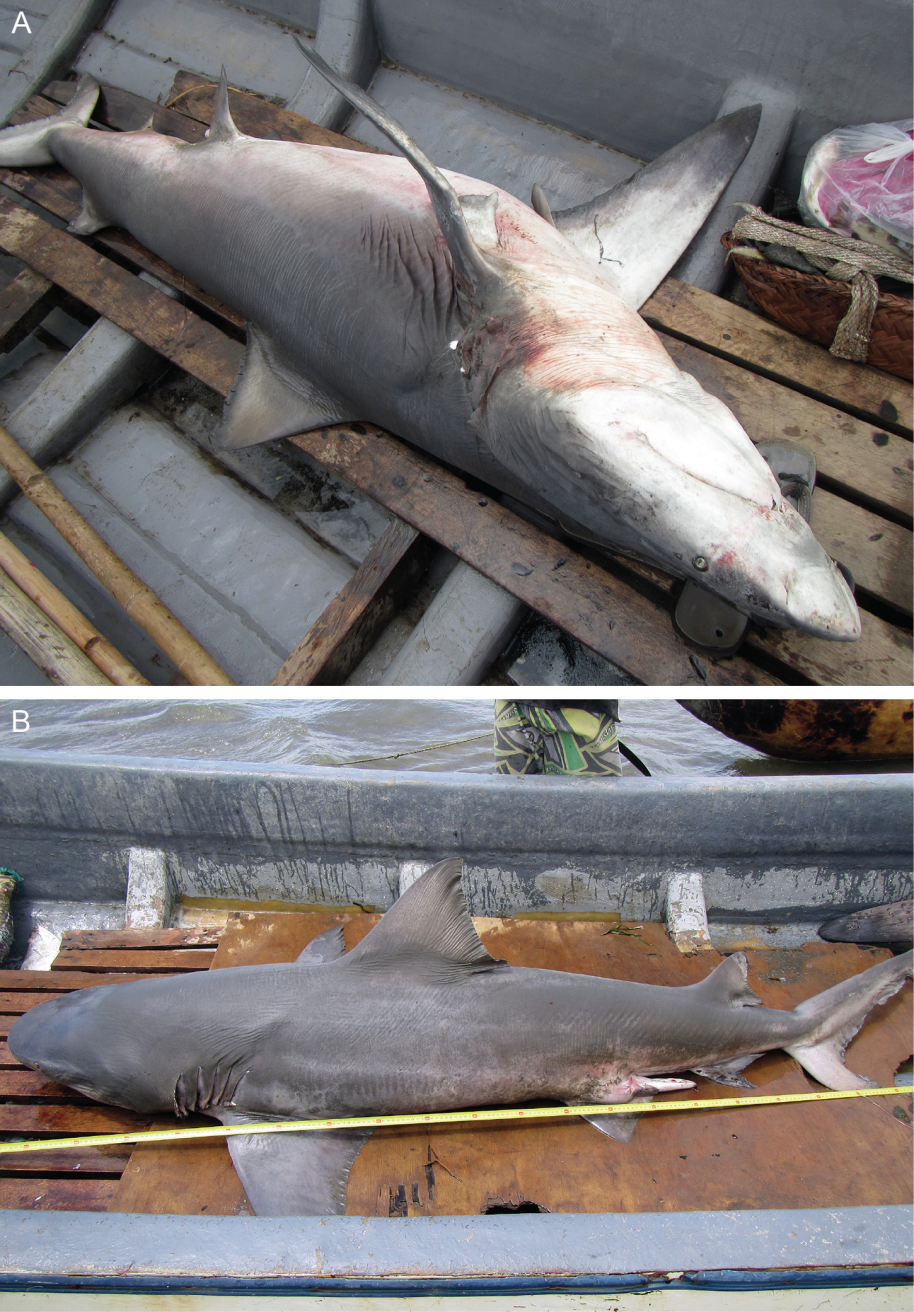
Freshly caught adult males of *Glyphis glyphis*. (A) estimated length 251–256 cm, caught 3^rd^ Nov. 2014; (B) ~228 cm TL, caught 13 November 2014.

**Fig 6:**
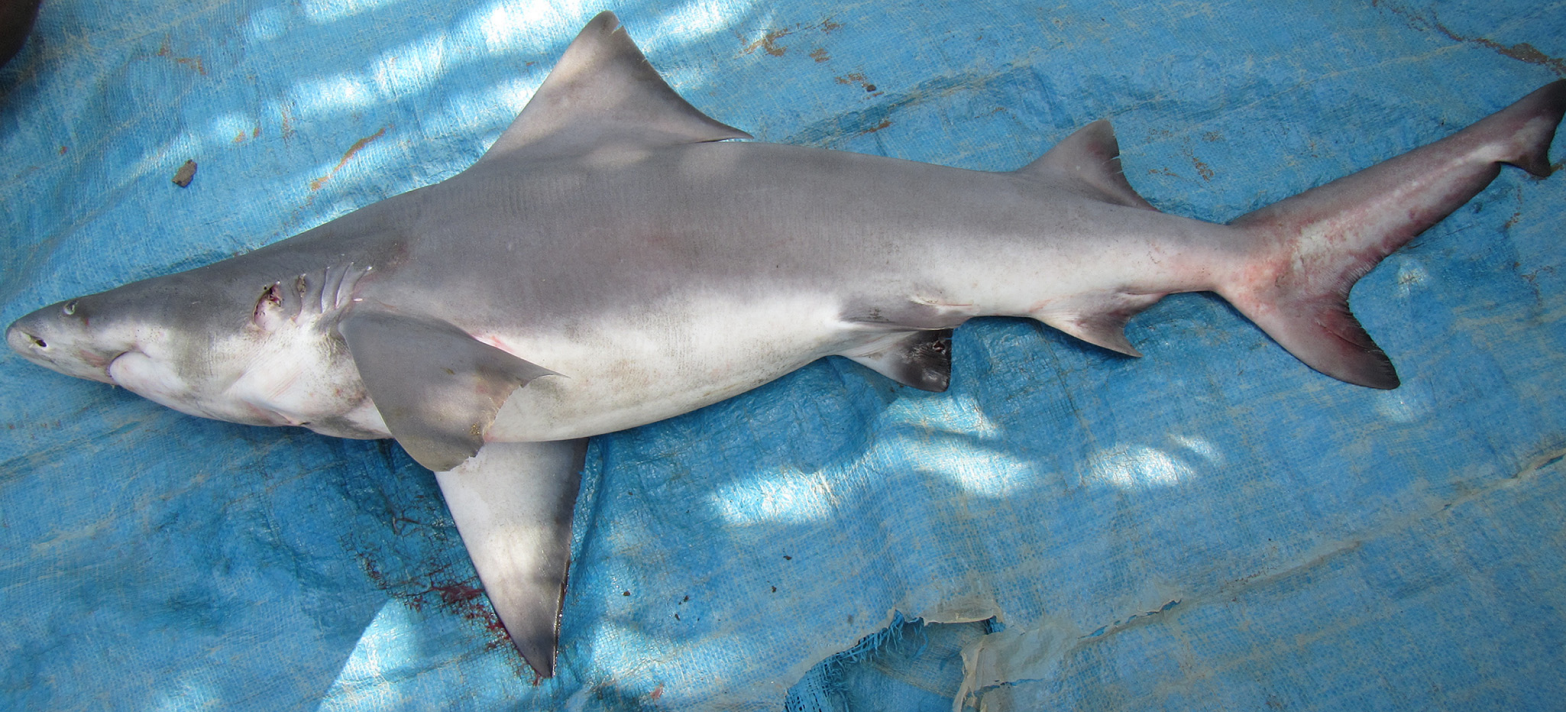
Freshly caught specimen of *Glyphis garricki*. Estimated length 100–105 cm, caught 6 November 2014.

The village chairman at Katatai was left with a camera to record any other sharks caught over the month of November 2014. In April 2015, images of three additional *Glyphis* specimens were sent through to the National Fisheries Authority in Port Moresby. These consisted of two adult male specimens of *G*. *glyphis* (Fig 5) and one unsexed specimen of *G*. *garricki* (Fig 6). Genetic samples on Whatman FTA Elute™ cards were also received for these specimens.

### Morphology

The first dorsal fin of the first *G*. *garricki* specimen (Fig 4) and the adult female *G*. *glyphis* (Fig 2A), and the left pectoral fin of the adult female *G*. *glyphis* (Fig 2E) were measured. For both pectoral and first dorsal fins, their length, anterior margin length, posterior margin length, height, and base length, were measured. One of the adult male *G*. *glyphis* (Fig 5B) and the *G*. *garricki* images supplied by the Katatai village chairman included the tape measure allowing total length to be estimated. For the second adult male *G*. *glyphis* (Fig 5A), the tape measure was only shown in the pectoral fin images. For both adult male specimens, the pectoral-fin length, anterior margin and posterior margin were photographed with the tape measure allowing an accurate size to be recorded for each. Fin measurement methodology follows [8]. In order to estimate a total length for the individual *G*. *garricki* for which only the dried dorsal fin was available, and for the adult female and one adult male of *G*. *glyphis*, the measurements provided in [4] for individuals of known total length were used. For *G*. *glyphis*, the dried type specimen measurements were excluded due to its poor condition. The estimated lengths obtained for *G*. *glyphis* using this data were compared with estimates based on the photographed adult male specimen supplied by the Katatai village chairman from which a total length and three pectoral fin measurements were obtained.

### Dentition

The dentition of the *G*. *glyphis* specimen was described and compared with the species redescription [4] which was based on specimens less than 1.8 m TL. During preparation of the jaw, a number of fish and stingray spines were removed. The dentition of two adult specimens of *G*. *garricki* (CSIRO H 6173–01 and CSIRO H 6635–01) was used for comparative purposes. The description of the adult female *G*. *glyphis* dentition and comparison with adult *G*. *garricki* are provided in S1 Appendix.

### Genetic analyses

Mitochondrial (mtDNA) genes are routinely used for species delineation [9,10]. Here we used three mtDNA genes, alongside morphological and dentition investigations for species determination. DNA barcoding enables the recognition and/or discrimination of an individual’s species identity based on short, relatively conserved gene fragments. Here we chose to use 16S rRNA (*16S*), cytochrome c oxidase subunit I (*COI*) and NADH dehydrogenase subunit 2 (*NADH2*) as DNA barcodes. *COI* is one of the most commonly accepted fragments for metazoan species discrimination [11]. There are a large number of *16S* sequences in GenBank and, due to its relatively slow rate of evolution, *16S* is often used for phylogenetic reconstructions and comparative purposes. Additionally, an ongoing Chondrichthyan Tree of Life (CToL) project led by one of us (GN) at the College of Charleston has primarily used the *NADH2* gene for species delineation as a first step towards collecting genomic scale information. The molecular analyses and barcoding were undertaken in two laboratories—the CSIRO marine laboratories in Australia (*16S* and *COI*) and the Hollings Marine Laboratory in Charleston, USA (*NADH2*). The specific methodology for the *16S* and *COI* analyses are provided in S1 Text.

## Results

### Genetic analyses

The *G*. *garricki* partial *16S* gene sequence (Accession number KR703623) was 547 base pairs (bp) in length while 562 bp (Accession number KR703625) of the *COI* gene was sequenced from the same individual. In the *G*. *glyphis* individual, 562 bp (Accession number KR703622) and 631 bp (Accession number KR703624) of the *16S* and *COI* genes respectively were successfully sequenced. When blasted against sequences in GenBank, there was 100% pairwise identity of the *G*. *garricki* sequences to Accession Number KF646786 (*Glyphis garricki* complete mitochondrial genome, [12]). Additionally, we observed 100% pairwise identity of the *G*. *glyphis* sequences to Accession Number KF006312 (*Glyphis glyphis* complete mitochondrial genome, [13]) and to the 93 *G*. *glyphis* whole mitochondrial genomes from Australia (Accession Numbers KM100613–KM100704) that were recently sequenced by [14].

**Fig 7:**
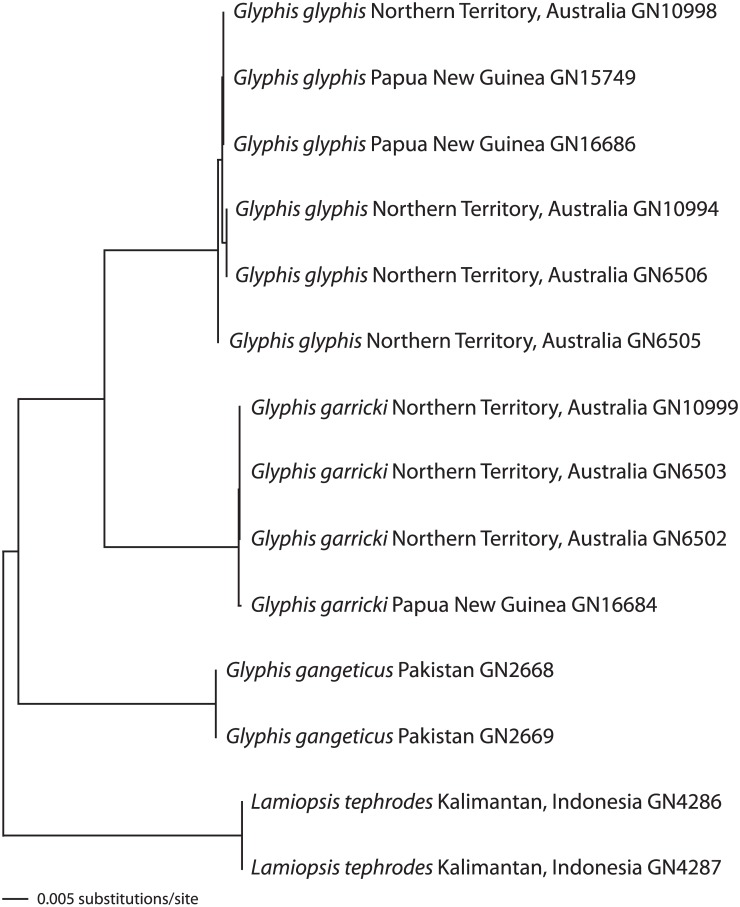
Molecular species identification using the mitochondrial *NADH2* gene. Neighbour-joining tree of *NADH2* sequences, estimated using the Kimura 2 parameter distance model of molecular evolution, of *Glyphis garricki*, *Glyphis glyphis*, *Glyphis gangeticus* and *Lamiopsis tephrodes*. The species identifications of samples GN15749 and GN16686 are confirmed as *G*. *glyphis* and sample GN16684 is confirmed as *G*. *garricki*.

Each of the three gene fragments (*COI*, *16S* and *NADH2*) yielded concordant identification of the two species. The tree resulting from the neighbour-joining analysis of the *NADH2* data derived from two of the three PNG *G*. *glyphis* specimens and one of the PNG *G*. *garricki* specimens, together with previously available sequences of other representatives of *G*. *glyphis* and *G*. *garricki*, another nominal congener *G*. *gangeticus* and an outgroup species *Lamiopsis tephrodes*, is shown in Fig 7. The two PNG specimens of *G*. *glyphis* (labelled GN15749 and GN16686 in Fig 7) cluster within the clade of *G*. *glyphis* sequences derived from individuals sampled in Australia. The PNG *G*. *garricki* specimen (labelled GN16684 in Fig 7) clusters within a separate clade comprised of *G*. *garricki* sequences that were also derived from Australian specimens.

Additionally, following FTA Elute extraction (SA pers. comm.) and gene sequencing using the same mtDNA genes as outlined above, the three FTA Elute genetic samples of the specimens caught by the Katatai village in November 2014 were identified as *Glyphis*; two adult male *G*. *glyphis* and a *G*. *garricki*.

### Estimation of total length

Using the first dorsal fin measurements of the types of *G*. *garricki* with known total lengths (see [4]), the dried *G*. *garricki* first dorsal fin was from a specimen estimated to be between 100 and 127 cm total length (TL). The total fin length, anterior margin length and base length produced estimates of 100, 102 and 105 cm TL, respectively, while the height and posterior margin produced estimates of 119 and 127 cm TL, respectively. The second *G*. *garricki* specimen, photographed by the Katatai village chairman, was ~113 cm TL.

Using the pectoral fin measurements of the measured specimens of *G*. *glyphis* in [4] with known total lengths, the size of the adult female *G*. *glyphis* specimen from Katatai was estimated to be between 282 and 389 cm TL. Using the first dorsal fin measurements, its size was estimated to be between 237 and 304 cm TL. The larger estimates produced using the pectoral fin measurements is likely the result of the ontogenetic changes in relative pectoral fin sizes in *Glyphis* species. The pectoral fins of *Glyphis* species become proportionally larger as they grow. Since the morphometric data for *G*. *glyphis* in [4] is based on eight whole specimens between 59 and 145 cm TL, estimating total length of a far larger specimen from these measurements is difficult. Using the total length and corresponding pectoral-fin measurements for one of the photographed adult males from Katatai overcomes this problem as they are similar in size. This produced estimates of 256, 260 and 304 cm TL for the adult female, based on pectoral-fin length, anterior margin and posterior margin, respectively.

One of the adult male *G*. *glyphis* specimens photographed by the Katatai village chairman had a total length of ~228 cm, based on a tape measure included in the image provided. The second adult male, for which only pectoral-fin length, anterior margin and posterior margin were recorded, was estimated to have a total length of 251, 256 and 292 cm, respectively.

The estimates of total length from the first dorsal fin measurements of the adult female *G*. *glyphis* followed a similar pattern to those for *G*. *garricki*. The total fin length, anterior margin length and base length produced estimates of 241, 237 and 242 cm TL, respectively, while the height and posterior margin produced larger estimates of 268 and 304 cm TL, respectively. Similarly, estimates obtained from the pectoral-fin posterior margin measurements yielded far higher estimates. Since for the two *Glyphis* species, the first dorsal-fin length, anterior margin length and base length produced more consistent estimates of total length, these measurements are considered better estimates of total length than the height and posterior margin length. In addition for *G*. *glyphis*, pectoral-fin length and anterior margin produced better estimates of total length. Table 1 summarises the sizes and estimated sizes for the five *Glyphis* specimens using the five fin measurements considered to be the most informative. Following this, the estimated size of the one *G*. *garricki* and two *G*. *glyphis* specimens from which a total length was not obtained were 100–105, 237–260, and 251–256 cm TL, respectively.

**Table 1:** Summary information on the five Papua New Guinea *Glyphis* specimens. Date of observation, genetic sample id, location, sex, total length (TL; lengths estimated from fin measurements in parantheses), and key measurements of the first dorsal and pectoral fins (in cm) of the *Glyphis* specimens recorded from the Daru region.

	Date	Location and part	Genetic id	sex	TL (cm)	D1L	D1A	D1B	P1L	P1A
***G*. *garricki***										
1	25 Oct 2014	Daru—dried dorsal fin (field code 220358)	KR703623 (16S); KR703625 (COI)	unknown	(100–105)	18	15	12.6	–	–
2	6 Nov 2014	Katatai observer—whole	GN16684 (NADH2)	unknown	~113	–	–	–	–	–
***G*. *glyphis***										
1	23 Oct 2014	Katatai—jaws^a^ and fins (field code 220348)	KR703622 (16S); KR703624 (COI); GN15749 (NADH2)	pregnant female	(237–260)	44.2	33.8	31	37.1	55.3
2	3 Nov 2014	Katatai observer—whole		adult male	(251–256)	–	–	–	33	48.5
3	13 Nov 2014	Katatai observer—whole	GN16686 (NADH2)	adult male	~228	–	–	–	30.2	44

Date of observation, genetic sample id, location, sex, total length (TL; lengths estimated from fin measurements in parantheses), and key measurements of the first dorsal and pectoral fins (in cm) of the *Glyphis* specimens recorded from the Daru region.

^a^jaws cleaned, dried and retained in the Australian National Fish Collection (CSIRO H 7670–01)

### Reproductive insights

The female *G*. *glyphis* specimen was a pregnant female. The fishers who caught the shark reported that it contained one fully developed pup (~65 cm TL) which was released alive. It is likely that more pups were present in the litter but were aborted whilst tangled in the gill net. The fishers reported that they have caught pregnant females (containing either six or seven pups) of this species previously, including in May and June.

### Dietary insights

**Fig 8:**
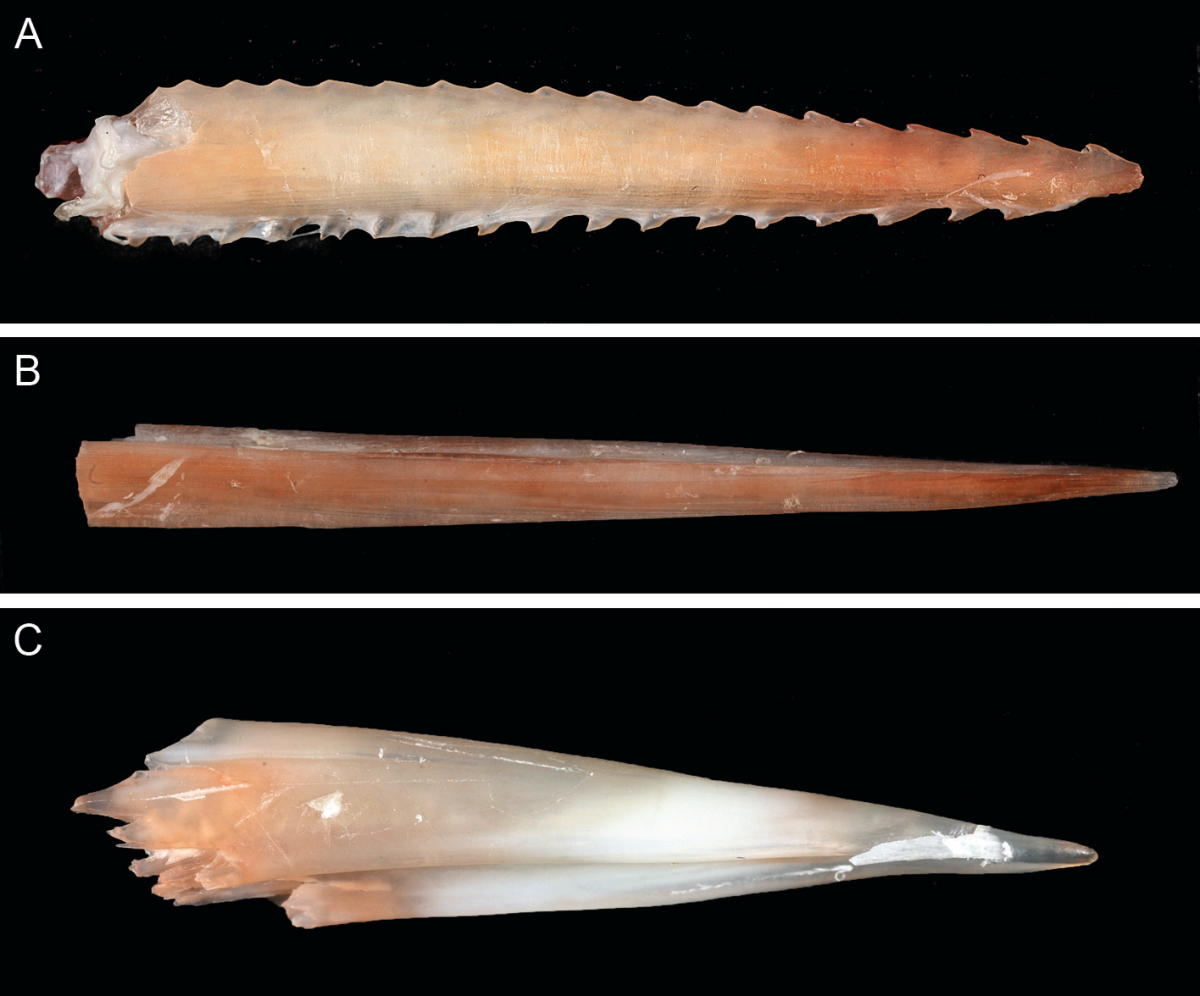
Spines from the connective tissue of the *Glyphis glyphis* jaw. Examples of spines found during dissection of the adult female jaw: (A) a stingray spine; (B) and (C), bony fish spines.

Although the fishers reported that the stomach of the *G*. *glyphis* specimen was empty, some insights into the diet of this species can be ascertained from spines present in the jaw. A single stingray spine (Fig 8A) and a large number of bony fish spines (e.g. Fig 8B and 8C) were found when removing connective tissue around the jaw. The majority of fish spines were well embedded in the cartilage. This suggests that bony fish and probably stingrays are likely to be important dietary items for adult *G*. *glyphis*. Attempts to extract DNA from the spines was successful, but when sequenced (*16S*) only the predator (*G*. *glyphis*) and not prey DNA was recovered.

## Discussion

### Genetic analyses

Mitochondrial markers have previously been used to discriminate between *G*. *garricki* and *G*. *glyphis* in northern Australia [15], and in the current study, the morphological and dentition studies are strongly supported by the sequencing results of the three genetic markers. The mtDNA sequences from *16S*, *COI* and *NADH2* confirm that the fin sample obtained from Philo Marine Ltd. in Daru in October 2014 was from a *G*. *garricki* individual and the muscle sample (from the village of Katatai) collected in the same month was from a *G*. *glyphis* individual. Three further individuals of *Glyphis* recorded by the Katatai fishers in November 2014 were confirmed to consist of two *G*. *glyphis* and one *G*. *garricki*.

### Life history implications

The biology of *Glyphis* species remains very poorly known [5], which is of concern given the conservation status of these species. Life history data such as fecundity and age is essential in understanding a species’ biological productivity and hence ability to sustain exploitation or to recover from over-exploitation [16,17]. The records of *G*. *garricki* and *G*. *glyphis* documented in this study represent the first confirmed records of these two species in PNG since the 1960s and 1970s. Despite the limited material available from the PNG specimens, these surveys have resulted in important information allowing more of the life history of these rare species to be pieced together, particularly that of *G*. *glyphis*. Of significance is the fact that the PNG specimens represent the first records of adult male and female *G*. *glyphis* anywhere within the species range. The previously reported maximum size for *G*. *glyphis* (175 cm TL; [5]) has been greatly surpassed here with a best estimate of 260 cm TL based on conversion of fin measurements. As previously postulated [4,5], *G*. *glyphis* is here confirmed as a large carcharhinid species, similar to *G*. *garricki*, which has been recorded to 251 cm TL in Australia [5].

Across all *Glyphis* species, there is only a single previously examined pregnant individual, a *G*. *garricki* from northern Australia with 9 pups [5]. Anecdotal reports from the Katatai village fishers of pregnant *G*. *glyphis* containing 6–7 pups provides the first estimate of litter size in that species. This highlights a probable low productivity, especially given that the reproductive cycle may be biennial as suggested for *G*. *garricki* [5], and which is regularly the case for medium-large sized carcharhinids [18]. The reported size of a fully-developed *G*. *glyphis* pup from the captured female (~65 cm TL) is consistent with the size at birth from northern Australia (50–65 cm TL; [5]). The capture of this pregnant female in October is also consistent with the timing of parturition in the Adelaide River, Australia [5]. It would thus appear that in both PNG and northern Australia parturition precedes the onset of the monsoonal wet season. The transition between the dry and wet seasons is a period of re-connectivity of aquatic environments enabling the movement of biota [19], potentially increasing prey availability for neonate sharks.

The capture of the adult *G*. *glyphis* specimens in coastal marine waters also provides important insights into the habitat use of this species. Records of *G*. *glyphis* from northern Australia are restricted to juveniles and subadults from tidal rivers, typically in fresh and brackish water. These records from marine water provide strong circumstantial evidence that coastal marine waters are an important habitat for adult *G*. *glyphis*.

A limited number of *G*. *glyphis* stomachs examined from Queensland, Australia contained teleost fish remains (particularly catfishes) as well as freshwater prawns (*Macrobrachium rosenbergii*) [20]. All previously examined sharks have been juveniles, and the presence of a stingray spine and many bony fish spines in the jaws of the adult female *G*. *glyphis* from PNG suggests that its diet is probably fish dominated. This also supports the benthic feeding habits of *G*. *glyphis* as suggested by the above-mentioned prey items [20].

### Conservation implications

These are the first documented records of *Glyphis* species in PNG waters since the 1960s and 1970s. Documenting their persistence in PNG improves our understanding of the contemporary distributions of these species that are of high conservation concern. Analysis of the genetic population structure of *G*. *glyphis* in northern Australia based on the whole mitochondrial genome showed a high degree of population separation between the three river drainages in which it occurs, suggesting strong female philopatry [14]. It is therefore possible that a New Guinean population(s) could be genetically distinct from the Australia populations. Limited exchange of individuals between these regions could indicate reduced resilience for depleted populations. It can be noted that the *NADH2* sequences (obtained from specific mtDNA genes) in this study cluster within the Australian sequences already obtained, although sequencing the whole mitogenome could provide more resolution of genetic structure [14].

The fact that adult *G*. *glyphis*, a large apex predator, have thus far gone unnoticed highlights the rarity of river sharks which combined with their occurrence in remote, poorly-surveyed regions, have resulted in *Glyphis* species being some of the least known sharks. Knowledge from artisanal fishers, together with the adult specimens documented here, can form the basis of surveys to document the occurrence and habitat of adults, a basic requirement for considering which management options may be appropriate in PNG for these rare species of high conservation concern.

## Supporting Information

S1 Appendix Dentition of adult *Glyphis glyphis*. (PDF)

S1 Text Genetic methodology. (DOCX)
