# Supplementary material for: Rediscovery of the Threatened River Sharks, Glyphis garricki and G. glyphis, in Papua New Guinea
Source: PLoS One. 2015 Oct 7;10(10):e0140075. doi: 10.1371/journal.pone.0140075 (PMC4596488; doi:10.1371/journal.pone.0140075)
Supplement: S1 Appendix — (PDF) [file pone.0140075.s001.pdf]

S1 Appendix. Dentition of adult *Glyphis glyphis*.

Dentition of the adult female *G. glyphis*

Odontological meristics of adult female *G. glyphis* (CSIRO H 7670-01): 13–0–13/13–1–13 with functional tooth series averaging 1–2/1–2. Essential tooth file (row) distribution may be defined as follows (left and right quadrants of each jaw with identical distribution counts): upper jaw: medial-0, anterior files 1–3 (A1, A2, A3), lateral files 4–10 (L1, L2, L3, L4, L5, L6, L7), posterior files 11–13 (P1, P2, P3); lower jaw: medial files 1, anterior files 1–3 (A1, A2, A3), lateral files 4–9 (L1, L2, L3, L4, L5, L6), posterior files 10–13 (P1, P2, P3, P4). Dignathic heterodonty strongly evident.

Upper jaw with gradient monognathic heterodonty present in all tooth groups (Fig. 1A); no toothless space in upper jaw but medial and symphysial teeth absent with productive oral tissue present; upper teeth compressed, broadly triangular with gradient serrations present descending from coarse and well developed basally to fine apically (Fig. 2); distal and mesial shoulders somewhat pronounced in anterior and lateral files but less so posteriorly; distal and mesial root lobes very symmetrical with outer edges straight, variably spaced throughout series but without imbrication with adjacent tooth; upper anterolateral crowns with nearly perpendicular mesial and distal edges in files A1–L1 becoming convex mesially and concave distally from files L2 rearward; crowns of posterior files strongly oblique with a gradient descent to two semi-molariform teeth with weakly developed crowns.

Lower jaw with gradient monognathic heterodonty present in all tooth groups (Fig. 1B); a single file of very weakly developed, asymmetrically placed symphysial teeth present at centre of symphysis with conical crowns curving lingually; anterior and anterolateral teeth with non-compressed, conical crowns and deeply concave basal structure (Fig. 3); root lobes on A1 and A2 asymmetrical with mesial lobe slightly shorter than distal lobe, subsequently becoming more

symmetrical in more distally placed teeth; exterior of root lobes with rounded edges anteriorly, becoming straight on L1 rearward; root lobes with slight alternate basal imbrication present in A1–A3 but becoming more widely spaced without overlap from L1 to posteriors; crowns of anterior teeth elongated, not serrated basally and strongly tapered at mid-crown; cusps abruptly expand apically becoming hastate with a finely serrated cutting edge; teeth in L1, L2 and L3 files of both lower quadrants with low, weakly developed, lobate basal cusplets (basal lobes) on distal and mesial crown feet; basal lobes diminish in posterolateral teeth becoming absent on L4–P4 files; hastate apical expansion only slightly present in lateral files L1 and L2 and serrated cutting edge gradually expands along full length of cusps in subsequent lateral and posterior files; basal structures of lateral teeth more expanded and less arched than anterior files and continually expanding posteriorly; posterolateral crowns gradually become oblique and less developed with cusps of posterior files reduced to very low but non-molariform cutting edge.

Comparison of adult *G. glyphis* and *G. garricki* dentition

Odontologically, *Glyphis* may be broken down into two primary groups: hastate vs. non-hastate with consistent basal lobe formation. Taking into consideration its geographical range and placement within the hastate group, *Glyphis glyphis* is most likely to be confused with its closest congener *Glyphis garricki*. Both species possess primarily erect, triangular upper teeth and narrow, variably hastate lower teeth. However, the lower anterior teeth of the two adult specimens of *Glyphis garricki* used in this study for comparative purposes possess a much weaker display of apical expansion with far less tapering at mid-crown and noticeably less hastate tips. In addition to this, at the time of publication, no specimens of *Glyphis garricki* have been reported to possess consistent, strongly developed basal cusplets (lobes) on the crown feet of the lower jaw teeth. Although not documented, it is highly likely that *Glyphis garricki* specimens could in fact variably possess basal cusplets on the lateral teeth of the lower jaw.

Varying degrees of presence of this particular development have been found in *Glyphis* specimens of both hastate and non-hastate structure from the Bay of Bengal and may prove to be less diagnostic in *Glyphis* than originally thought. Nonetheless, it is an important feature when separating some specimens of the hastate group from non-hastate specimens with slightly large apical expansion as observed in some *Glyphis gangeticus*.

Taking this into consideration, the primary odontological features separating *Glyphis glyphis* from *Glyphis garricki* would be dental meristics and medial tooth development. Firstly, meristics would be primary in this separation with *Glyphis glyphis* having a much lower and consistent tooth count of 13–14/12–14 per quadrant versus a known count of 15–17/15–17 in *Glyphis garricki*, excluding medials in both summaries. This separation holds true as well with *gangeticus*-like specimens from Pakistan and the Bay of Bengal which tend to possess counts in the range of 14–18/14–18. Secondly, although both *G. garricki* and *G. glyphis* possess the development of medial teeth in the lower symphysis (less so in *G. glyphis*), only *G. garricki* has been verified as having strongly developed upper medials, sometimes as many as two. *Glyphis glyphis* very rarely has any such development, having only been noted so far in one immature female from Port Romilly, PNG (LWF-E-218). This specimen was found to have one weakly developed medial tooth in the upper jaw.

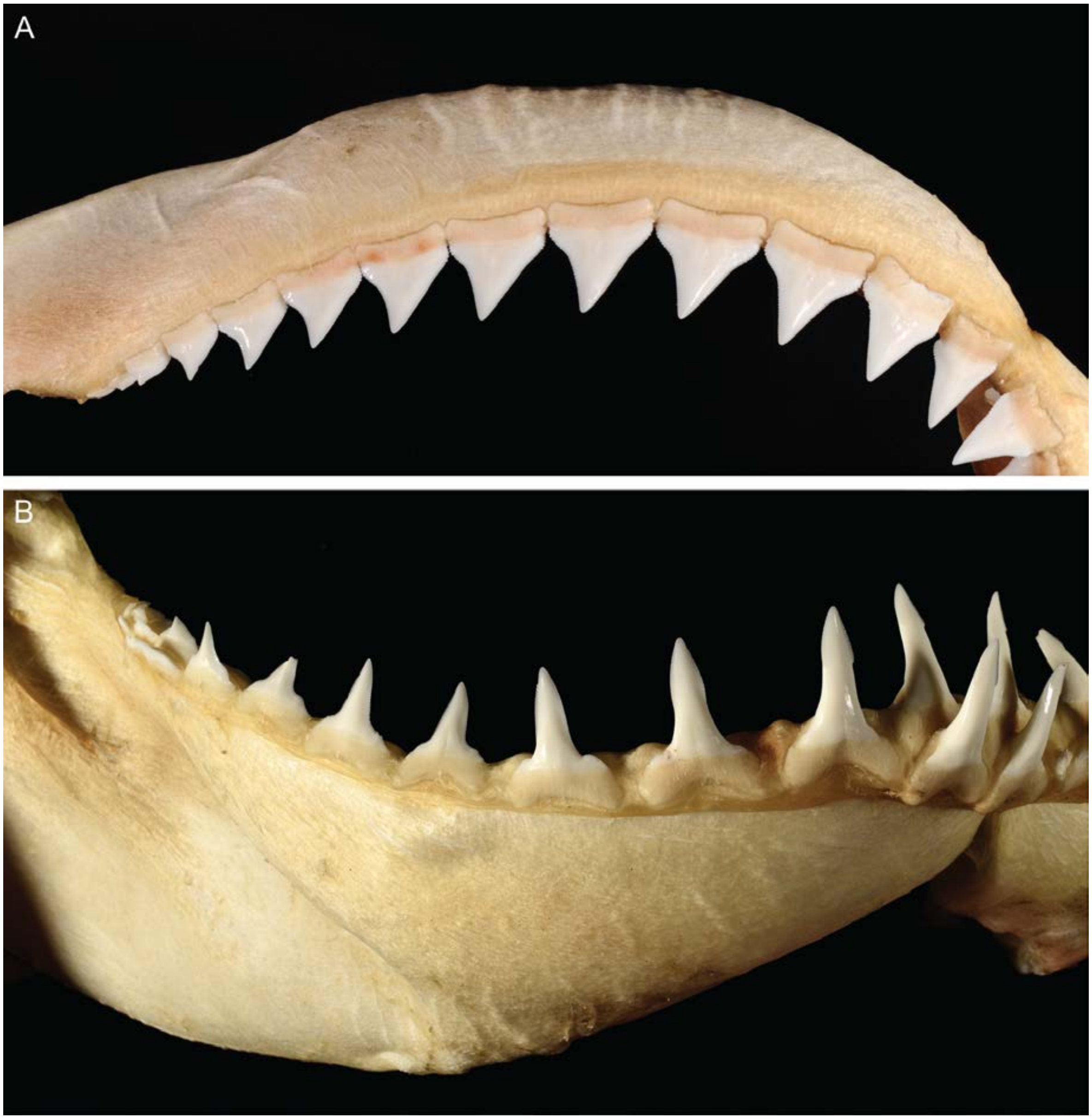

Figure 1 **Upper and lower teeth of *Glyphis glyphis***

Adult female CSIRO H 7670-01: (A) upper teeth on right side of jaw; (B) lower teeth on right side of jaw.

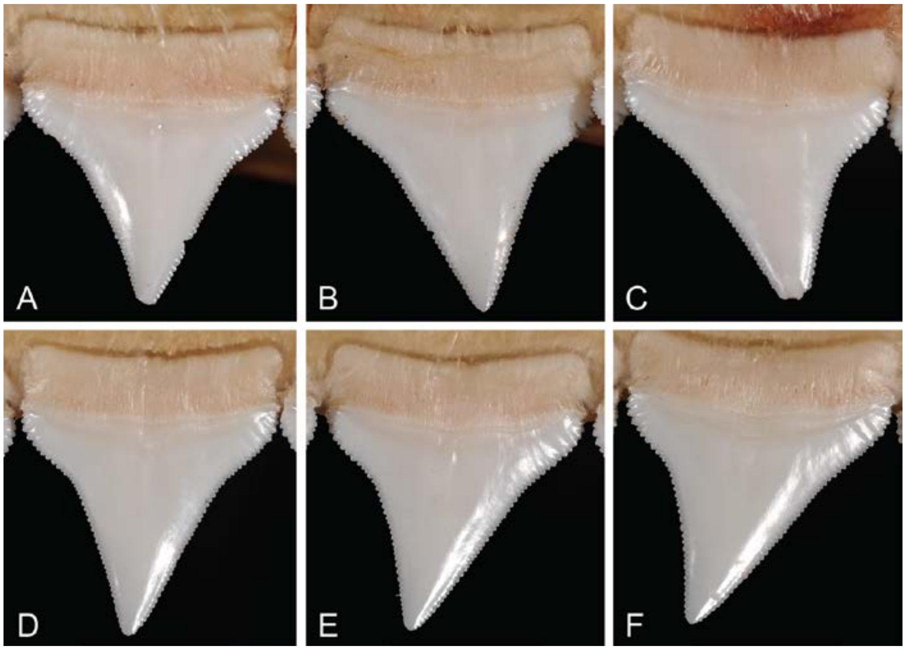

Figure 2 **Upper anterior and anterolateral teeth of *Glyphis glyphis***

Adult female (CSIRO H 7670-01): (A-C) left side; (D-F) right side; (A) and (D) 3rd tooth, A3; (B) and (E) 4th tooth, L1; (C) and (F) 5th tooth, L2.

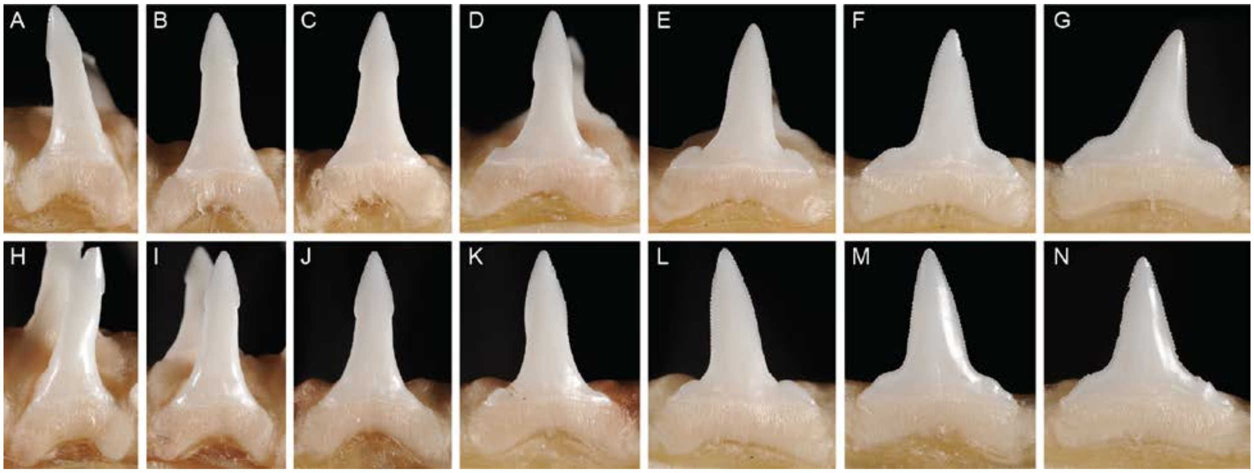

Figure 3 **Lower anterior and anterolateral teeth of *Glyphis glyphis***

Adult female (CSIRO H 7670-01): (A-G) left side; (H-N) right side; (A) and (H) 1st tooth, A1; (B) and (I) 2nd tooth, A2; (C) and (J) 3rd tooth, A3; (D) and (K) 4th tooth, L1; (E) and (L) 5th tooth, L2; (F) and (M) 6th tooth, L3; (G) and (N) 7th tooth, L4.
