# Supplementary material for: Rediscovery of the Threatened River Sharks, Glyphis garricki and G. glyphis, in Papua New Guinea
Source: PLoS One. 2015 Oct 7;10(10):e0140075. doi: 10.1371/journal.pone.0140075 (PMC4596488; doi:10.1371/journal.pone.0140075)
Supplement: S1 Text — (DOCX) [file pone.0140075.s002.docx]

**S1 Text. Genetic methodology.**

***16S* and *COI***

Genomic DNA from the *G. glyphis* muscle sample and *G. garricki* dried fin sample were extracted using the Wizard® SV Genomic DNA Purification system (Promega, USA) with starting material of approximately 0.25 g. Tissue extractions were undertaken using SV minicolumns following the manufacturer’s instructions with modifications including digestion with 400 *μ*g Proteinase K and precipitation of DNA in 160 *μ*l nuclease free water. DNA quality and quantity was determined on a Nanodrop 8000 (Thermo Scientific, USA) UV-Vis Spectrophotometer. Working volumes of DNA were stored at 4°C while archival samples are stored at -80°C.

The mitochondrial gene regions *16S* and *COI* were amplified in the genomic DNA using the universal 16SarL and 16SbrH primers [1] and COI FISH-BCL and FISH-BCH primers [2], respectively. Polymerase chain reactions (PCRs) were carried out in 25 *μ*l using GoTaq® Green Master Mix (Promega, USA), Bovine Serum Albumin (Promega, USA), 10 µM primers and DNA quantities of between 10 and 15 ng. PCRs were performed in an Applied Biosystems GeneAmp® PCR System 9700 (Life Technologies, Thermo Fisher Scientific, USA) with cycling conditions of 94°C × 3 mins, 35 cycles of 94°C × 1 min, 54°C/50°C (*16S*/*COI* respectively) × 1 min 30 sec, 72°C × 1 min, with a final extension of 72°C × 10 mins. PCR products were visualised on 2.5% TAE agarose gels and fragments cleaned using an Agencourt AMPure XP PCR purification kit (Beckman Coulter, Australia) according to the manufacturer’s instructions. PCR products were sequenced bi-directionally using the same primers as in the original PCR, BigDye® Terminator v3.1 Cycle sequencing kit (Life Technologies) and the same annealing temperature (for each gene) × 5 sec across 25 cycles. Cycle sequenced products were cleaned using the CleanSEQ kit (Beckman Coulter) according to the manufacturer’s instructions and run on an ABI 3130 xl AutoDNA sequencer (Life Technologies). Forward and reverse sequences (per gene fragment) were assembled into consensus sequences in Geneious® R8.1.4 (Biomatters Ltd Auckland, New Zealand; <http://www.geneious.com>). Consensus sequences were aligned using the MAFFT module (within Geneious). Sequence identity was confirmed by using the BLAST module in Geneious (<http://blast.ncbi.nlm.nih.gov/Blast.cgi;Megablast>) against GenBank (<http://www.ncbi.nlm.nih.gov/genbank/>). The four consensus sequences generated here were deposited in GenBank under the following accession numbers (G. garricki 16S rRNA Accession Number KR703623 and G. glyphis 16S rRNA Accession Number KR703622; *G. garricki* *COI* Accession Number KR703625 and *G. glyphis* *COI* Accession Number KR703624).

**NADH2**

DNA was extracted using the E.Z.N.A Tissue DNA Kit (Omega Bio-Tek Inc., Norcross, GA) following the manufacturer’s instructions. Extracted total DNA was quantified visually using a 2% agarose gel and stored at –20°C until used for PCR amplification of the *NADH2* region. A single set of universal primers [3] designed to bind to the ASN and ILE tRNA regions of the mitochondrial genome were used to amplify the target fragment. PCR reactions were carried out in 25 *μ*l containing 0.3 *µ*M primers, 2.5 mM MgCl_2_, 200 *µ*M each dNTP, 10X *Ex Taq* buffer (20 mM Tris-HCl pH 8.0, 100 mM KCl, 0.1mM EDTA, 1mM DTT, 0.5% Tween20, 0.5% Nonidet P-40, 50% Glycerol), 0.25 U *TaKaRa Ex* Taq (Takara, Mountain View, California), and 50–100 ng DNA. PCRs were performed in an Eppendorf Mastercycler with cycling conditions of 94°C × 3 mins; 35 cycles of 94°C × 30 s, 48°C × 30 s, 72°C × 1 min 30 s; with a final extension of 72°C × 10 mins. PCR products were purified with ExoSAP-IT (USB, Cleveland, Ohio), and bi-directionally sequenced using the same *NADH2* primers as above and BigDye^®^ 3.1 sequencing chemistry on an ABI 3730*xl* AutoDNA sequencer (Life Technologies) at Retrogen Inc. Custom DNA Sequencing Facility (San Diego USA).

*NADH2* DNA sequences were translated to amino acids and aligned against reference sequences of *G. glyphis* and *G. garricki* from Northern Australia, reference sequences of *G. gangeticus* from India and Pakistan, and sequences of *L tephrodes* from Indonesia using the MAFFT module within Geneious® Pro v. 6.1.7 (Biomatters). The aligned amino acid sequences were back translated, in frame, to yield a nucleotide alignment that was 1044 bp in length. A neighbour-joining analysis of the aligned nucleotide sequences was conducted using a Kimura 2 parameter distance model of molecular evolution using the software package PAUP*4.0 version a 145.

**References**

1. Palumbi S, Martin A, Romano S, McMillan WO, Stice L, Grabowski G. The simple fools guide to PCR version 2.0. Honolulu, HI: Department of Zoology and Kewalo Marine Laboratory, University of Hawaii; 1991.
2. Baldwin CC, Mounts JH, Smith DG, Weigt LA. Genetic identification and colour descriptions of early life-history stages of Belizean *Phaeoptyx* and *Astrapogon* (Teleostei: Apogonidae) with comments on identification of adult *Phaeoptyx*. Zootaxa. 2009;2008: 1–22.
3. Naylor GJP, Ryburn JA, Ferigo O, Lopez A. Phylogenetic relationships among the major lineages of modern elasmobranchs. In: Hamlett WC (ed) Reproductive biology and phylogeny of Chondrichthyes: sharks, batoids and chimaeras. Science Publishers, Enfield; 2005. pp 1–25.
